# TRDMT1 exhibited protective effects against LPS‐induced inflammation in rats through TLR4‐NF‐κB/MAPK‐TNF‐α pathway

**DOI:** 10.1002/ame2.12221

**Published:** 2022-04-27

**Authors:** Zhengguang Li, Xiaolong Qi, Xu Zhang, Lei Yu, Lijuan Gao, Weining Kong, Wei Chen, Wei Dong, Lijun Luo, Dan Lu, Lianfeng Zhang, Yuanwu Ma

**Affiliations:** ^1^ Key Laboratory of Human Disease Comparative Medicine, National Health Commission of China (NHC) Institute of Laboratory Animal Science, Chinese Academy of Medical Sciences, Peking Union Medicine College Beijing China; ^2^ National Human Diseases Animal Model Resource Center and Beijing Engineering Research Center for Experimental Animal Models of Human Critical Diseases Institute of Laboratory Animal Science, Peking Union Medicine College, Chinese Academy of Medical Sciences Beijing China; ^3^ Neuroscience Center Chinese Academy of Medical Sciences Beijing China

**Keywords:** inflammation, knockout rat, TLR4 pathway, TNF‐α, *Trdmt1*

## Abstract

**Background:**

Inflammation is a complex physiological and pathological process. Although many types of inflammation are well characterized, their physiological functions are largely unknown. tRNA aspartic acid methyltransferase 1 (TRDMT1) has been implicated as a stress‐related protein, but its intrinsic biological role is unclear.

**Methods:**

We constructed a *Trdmt1* knockout rat and adopted the LPS‐induced sepsis model. Survival curve, histopathological examination, expression of inflammatory factors, and protein level of TLR4 pathway were analyzed.

**Results:**

*Trdmt1* deletion had no obvious impact on development and growth. *Trdmt1* deletion slightly increased the mortality during aging. Our data showed that *Trdmt1* strongly responded in LPS‐treated rats, and *Trdmt1* knockout rats were vulnerable to LPS treatment with declined survival rate. We also observed more aggravated tissue damage and more cumulative functional cell degeneration in LPS‐treated knockout rats compared with control rats. Further studies showed upregulated TNF‐α level in liver, spleen, lung, and serum tissues, which may be explained by enhanced p65 and p38 phosphorylation.

**Conclusions:**

Our data demonstrated that *Trdmt1* plays a protective role in inflammation by regulating the TLR4‐NF‐κB/MAPK‐TNF‐α pathway. This work provides useful information to understand the TRDMT1 function in inflammation.

## Graphical abstract

*Trdmt1* knockout rats were more vulnerable to LPS treatment with increased mortality and more aggravated tissue damage including noticeably thickened alveolar walls in lung tissues compared with WT. Elevated TNF‐α level production in liver, spleen, lung, and serum tissues were observed during LPS‐induced inflammation.

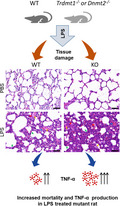

## 1 INTRODUCTION

Inflammation is a complex physiological and pathological process triggered by external and internal stimuli and conditions, such as infection, tissue injury, and aging.^1^, ^2^ Considerable achievements have been made in understanding the pathological process involved in the inflammatory response to infection or tissue injury. Dysregulation of inflammation during infection or tissue injury is a progressive and complex progress with unclear mechanism. Lipopolysaccharide (LPS), also known as endotoxin, is the main endogenous molecular component of the cell wall of Gram‐negative bacteria.^3^ LPS is usually used as an exogenous stimulus of inflammatory responses.^4^ LPS‐induced sepsis is a good tool to study inflammation progression.^5^, ^6^

tRNA aspartic acid methyltransferase 1 (TRDMT1), also known as DNMT2, was initially identified as a member of the DNA cytosine C5 methyltransferase (DNMT) family because it shares a conserved catalytic function domain with DNMT1.^7^ TRDMT1 is highly conserved among vertebrates.^8^ Evidence indicates that TRDMT1 acts as a tRNA methyltransferase rather than a DNA methyltransferase.^9^, ^10^ TRDMT1 methylates tRNA^Asp‐GUC^, tRNA^Gly‐GCC^, tRNA^Val‐AAC^, tRNA^Glu‐CUC^, tRNA^Val‐CAC^, and tRNA^Gln‐CUG^ at the C5 position of C38 close to the anticodon.^11^, ^12^ This tRNA methylation function has been associated with tRNA stability and protein synthesis.^13^ TRDMT1 has been implicated in regulation of brain development, myocardial hypertrophy, transgenerational inheritance, hematopoietic stem cell (HSC) steady state, human immunodeficiency virus (HIV) infection, and drug sensitivity.^14^, ^15^, ^16^, ^17^, ^18^, ^19^, ^20^ Moreover, TRDMT1 plays an important role in resistance to stress, including oxidative stress, salt stress, and cellular senescence.^13^, ^19^, ^21^, ^22^, ^23^, ^24^, ^25^, ^26^ Even though TRDMT1 has been reported to be associated with longevity, the underlying mechanism is still unclear. Inflammation is a systemic and broad response to stress, and whether TRDMT1 plays a role in inflammation progression remains unknown.^26^, ^27^

Rats are indispensable laboratory animals to explore the human genome function by modeling disease processes. Rats differ from mice in terms of physiology, with some important traits that make rats a model of choice for complex human diseases. For example, rats share more immune characteristics with humans than mice do.^28^ The complement levels in rats and humans are comparable, whereas in most inbred mice they are very low.^29^, ^30^ In this context, we used rats to construct a *Trdmt1* gene knockout model and adopted LPS‐induced inflammation to study the function of *Trdmt1* in inflammation progression. Our data showed that *Trdmt1* deletion made rats more vulnerable to LPS treatment. The gene deletion was correlated with increased LPS‐induced death, and this phenotype can be largely explained by aggravated multiple organ damage with pronouncedly increased tumor necrosis factor‐alpha (TNF‐α) level. We further explored the underlying molecular mechanism of *Trdmt1* participating in regulation of inflammation progression in LPS‐induced inflammation.

## 2 MATERIALS AND METHODS

### 2.1 Animal

All rats were purchased from Beijing Hfk Bioscience, and housed in a specific pathogen‐free (SPF) facility. Rats were under a 12/12‐hours light cycle with free access to food and water. All experiment procedures in this study were reviewed and approved by the Institutional Animal Care and Use Committee of the Institute of Laboratory Animal Sciences, Chinese Academy of Medical Sciences and Peking Union Medical College (MYW19004).

### 2.2 DNA construction and sgRNA preparation

The single‐guide RNA (sgRNA) expression plasmid was constructed as reported previously.^31^ In brief, the synthesized paired oligos for sgRNAs were annealed and cloned into the pUC57‐sgRNA plasmid (#51132, Addgene). The oligos used in this study were purchased from Thermo Fisher and are listed in Table S1. The sgRNA expression plasmids were linearized by *Dra I*, purified by phenol‐chloroform, and used as template for transcription. The sgRNA transcription was performed using MEGAshortscript Kit (Ambion, AM1354) and purified using the MEGAclear Kit (Ambion, AM1908).

### 2.3 Generation of *Trdmt1* knockout rat

The fertilized eggs were collected and prepared for microinjection as described previously.^32^ In brief, the fertilized eggs were injected with a mixture of Cas9 protein and targeting sgRNAs with the concentration as reported previously.^33^ The injected zygotes were transferred to prepared pseudopregnant Sprague–Dawley (SD) rats.

### 2.4 Rat genomic DNA extraction and genotyping

Genomic DNA of newborn pups was extracted from the tails of 7‐day‐old rat using phenol‐chloroform and recovered by alcohol precipitation. The primer pair *Trdmt1*‐P1 and *Trdmt1*‐P2 was used to detect the knockout brand. The primer pair *Trdmt1*‐P3 and *Trdmt1*‐P4 was used to detect the WT brand. The PCR conditions were as follows: 95°C for 30 s; 95°C for 30 s, 60°C for 30 s, and 72°C for 90 s for 30 cycles. Twenty microliters reaction mixture was run on a 1.5% agarose gel, and the GoodView Nucleic Acid Stain (HGV‐II, sbs)‐stained products were visualized using Gel Image System (Tanon‐1600). All primers used for PCR are listed in Table S2.

### 2.5 Quantitative real‐time PCR

Total RNA from tissues and cells were extracted using TriQuick Reagent (Solarbio). Two micrograms of RNA samples was used for cDNA synthesis using PrimeScript RT reagent kit with a gDNA Eraser kit (Perfect Real Time) according the manufacturer's instructions. Real‐time PCR was performed with PerfectStart Green qPCR SuperMix (+Dye II) (Transgen) using QuantStudio^T^ 3 (Applied Biosystems) as follows: 95°C for 30 s; 95°C for 5 s and 60°C for 30 s for annealing/extension for 40 cycles. All primers used for real‐time PCR are listed in Table S3.

### 2.6 LPS‐induced inflammation in rats

Eight‐week‐old SD rats were injected intraperitoneally with 5 or 1 mg/kg LPS (L4391, Sigma). *Trdmt1* expression and animal survival rate were monitored. The rats treated with 1 mg/kg LPS were used for pathology and molecular detection.

### 2.7 Lung wet/dry (W/D) ratio

The isolated lung was rinsed with saline, and the excess water was absorbed with filter paper. Then, the lung was wrapped in tinfoil and weighed as W1. It was transferred to an incubator at 80°C for 24 hours. In the final hour, continuous measurement was taken 3 times and average recorded as W2. The lung W/D ratio was calculated as W1/W2.

### 2.8 Hematoxylin and eosin staining

Liver, spleen, lung, and kidney isolated from rats were preserved in 4% buffered formalin solution fixed, and then prepared for the paraffin sections (3–4 μm thickness) according to routine methods. All tissue sections were stained with hematoxylin and eosin (H&E) according to previous publications.^34^ The histopathological changes of multiple tissues were observed and analyzed under Pannoramic 250 FLASH and CaseViewer 2.3 (3D HISTECH).

### 2.9 Lung injury score

Lung injury score was assessed using the following scoring system: (a) alveolar congestion, (b) alveolar hemorrhage, (c) neutrophil infiltration, (d) thickness of alveolar wall/hyaline membrane formation (absent = 0, minor = 1, moderate = 2, severe = 3, critical = 4). The total lung injury score was the sum of the 4 criteria. Twenty high‐power random fields (400× total magnification) were scored per rat.

### 2.10 ELISA

Serum from each rat was collected to measure the TNF‐α level by ELISA kit (RTA00, R&D) according to the manual. Briefly, after adding 50 μL of assay diluent to each well, the serum samples were diluted at 1:100, and then 50 μL standard, control, or samples were added to different precoated wells and maintained at room temperature for 2 hours, followed by 5 washes, and then 100 μL of conjugate was added to each well, and the samples were incubated at room temperature for 2 hours. Then, 100 μL Substrate Solution was added to each well, and the samples were incubated at room temperature for 30 minutes. Finally, 100 μL of Stop Solution was added to each well, and the absorbance of 450 nm was recorded by a microplate reader (Multiskan FC with Incubator 51 119 100, Thermo) within 30 minutes.

### 2.11 Alveolar macrophage (AM) isolation, culture, and treatment

SD rats were anesthetized with 2% pentobarbital sodium (3 mL/kg) via intraperitoneal injection. The abdominal cavity was opened, then the thoracic cavity was opened through the mediastinum, and a small incision (<2 mm) was made on the trachea posterior to the larynx. A 1‐inch 22 G catheter was inserted and secured with a silk braided suture with a square knot. Ice‐cold BAL buffer (Ca^2+^‐ and Mg^2+^‐free PBS + 1 mM EDTA) was filled in a 5 mL syringe attached to the catheter and instilled slowly into the lungs. The lavage fluid was aspirated and collected in a 15 mL conical tube placed on ice to full. BAL fluid was centrifuged at 250*g* at 4°C for 10 minutes, and the supernatant was removed. AM was washed 3 times with precooled RPMI‐1640 medium and incubated in a 37°C humidified incubator with 5% CO_2_. AM was treated with 100 ng/mL LPS and analyzed by quantitative real‐time PCR.

### 2.12 Peritoneal macrophage (PM) isolation, culture, and treatment

SD rats were anesthetized with 2% pentobarbital sodium (3 mL/kg) via intraperitoneal injection. The abdominal cavity was opened with a small incision. The abdominal cavity was washed with 10 mL PBS with a 25 G needle. The peritoneal lavage fluid was centrifuged at 250*g* at 4°C for 10 minutes, and the supernatant was removed. PM was washed 3 times with precooled RPMI‐1640 medium and incubated in a 37°C humidified incubator with 5% CO_2_. PM was treated with 100 ng/mL LPS and analyzed by quantitative real‐time PCR.

### 2.13 Western blot

Total proteins from tissue were extracted on ice and prepared in 1× loading buffer. Proteins were separated by 10% SDS‐PAGE and transferred to nitrocellulose (NC) membranes. The membranes were blocked with 5% skimmed milk at room temperature for 1 hour, followed by incubation with primary antibody overnight at 4°C. Then the membrane was incubated with HRP‐conjugated secondary antibody at room temperature for 1 hour. Antigen complexes were visualized with ECL and analyzed using Image Lab (Bio‐Rad). Antibodies are listed in Table S7.

### 2.14 Statistical analysis

All data were analyzed by the GraphPad Prism 8.0 software and presented as the mean ± standard error of mean (SEM). Data with 2 groups or multiple groups were analyzed by 2‐tailed Student's *t* test or 2‐way analysis of variance (ANOVA). The survival rate was analyzed by log‐rank nonparametric test and shown as Kaplan–Meier survival curve. *P* < 0.05 was defined as statistically significant.

## 3 RESULTS

### 3.1 Generation of *Trdmt1* knockout rat

*Trdmt1* is conserved among vertebrates, but the gene function is confusing and controversial. Here we use rat, an indispensable animal model for illustrating the human biological progress through genetic modification and phenotype networks,^35^, ^36^ to investigate the gene function of *Trdmt1*.

**FIGURE 1:**
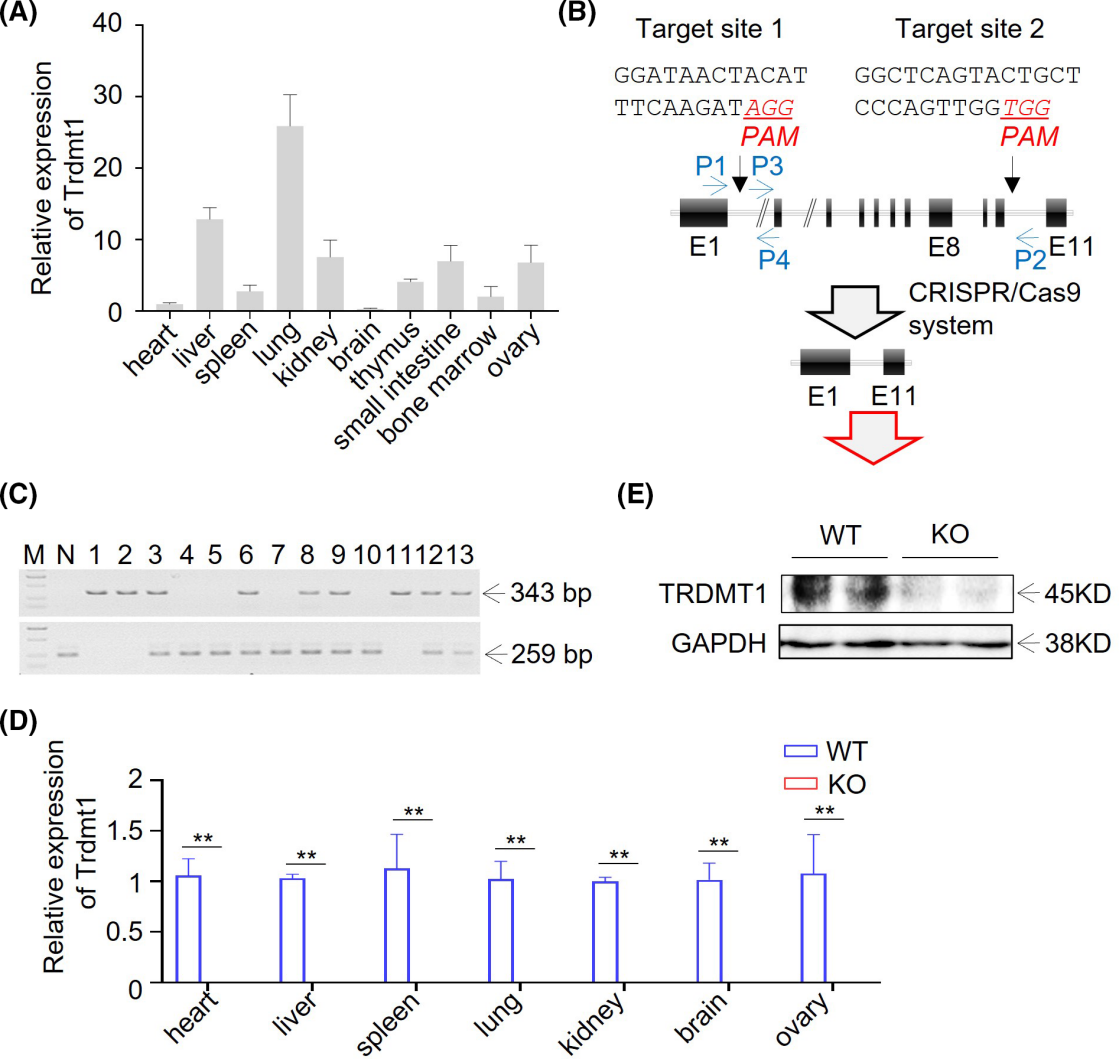
Generation of *Trdmt1* gene knockout rat using CRISPR/Cas9. A, Relative expression of *Trdmt1* in heart, liver, spleen, lung, kidney, brain, thymus, small intestine, bone marrow, and ovary tissues measured using real‐time PCR (*n* = 3). B, Schematic overview of CRISPR/Cas9‐mediated *Trdmt1* gene deletion. The sgRNA targeting sites are shown. The protospacer adjacent motifs (PAMs) of sgRNA targeting sites are underlined in red and italic. Oligos used for sgRNAs preparation are listed in Table S1. Primers used for genotyping of *Trdmt1* knockout rats are shown in blue and listed in Table S2. E, exon. C, PCR amplicon of *Trdmt1* targeting locus. P1/P2 primer pair located outside of the sgRNA targeting sites and used for deleted DNA fragment detection. The P3/P4 primer pair located between the 2 sgRNA targeting sites and used for wild‐type DNA fragment detection. All primers used are listed in Table S2. M, marker for DL2, 000 (TaKaRa). N, negative control (wild type). D, The relative expression of *Trdmt1* in heart, liver, spleen, lung, kidney, brain, and ovary tissues confirmed in *Trdmt1* knockout rats using real‐time PCR (*n* = 3). E, Protein level of TRDMT1 in lung of *Trdmt1* knockout rats. WT, wild‐type rats; KO, *Trdmt1* knockout rats. Mean ± SEM; *P < 0.05, **P < 0.01

Firstly, we examined the gene expression of *Trdmt1* in different tissues of Sprague–Dawley (SD) rats by quantitative real‐time PCR. The data show that *Trdmt1* was mainly expressed in liver and lung (Figure 1A). To study in vivo function, we constructed a *Trdmt1* gene knockout rat using CRISPR/Cas9 system as described previously.^31^ In brief, 2 sgRNAs were designed, one target site located in intron 1 and the other in intron 10, to produce fragment deletion from exon 2 to exon 10 (Figure 1B, Table S1). After sgRNA and Cas9 protein mixture microinjection and embryo transplantation, we obtained 7 rats, one with DNA fragment deletion detected by target primer pairs (Table S2). Then, we crossed this F_0_ rat with wild‐type (WT) SD rat and found the modification was germline transmittable (Figure 1C, Table S2). The target modification with a 28, 727 bp deletion was confirmed using Sanger sequencing. Further quantitative real‐time PCR results validated the expression of *Trdmt1* knockout rat in various tissues (Figure 1D, Table S3). Moreover, The TRDMT1 protein deletion was further confirmed by western blot in lung tissue (Figure 1E).

### 3.2 Loss of *Trdmt1* slightly affected development and growth in rats

**FIGURE 2:**
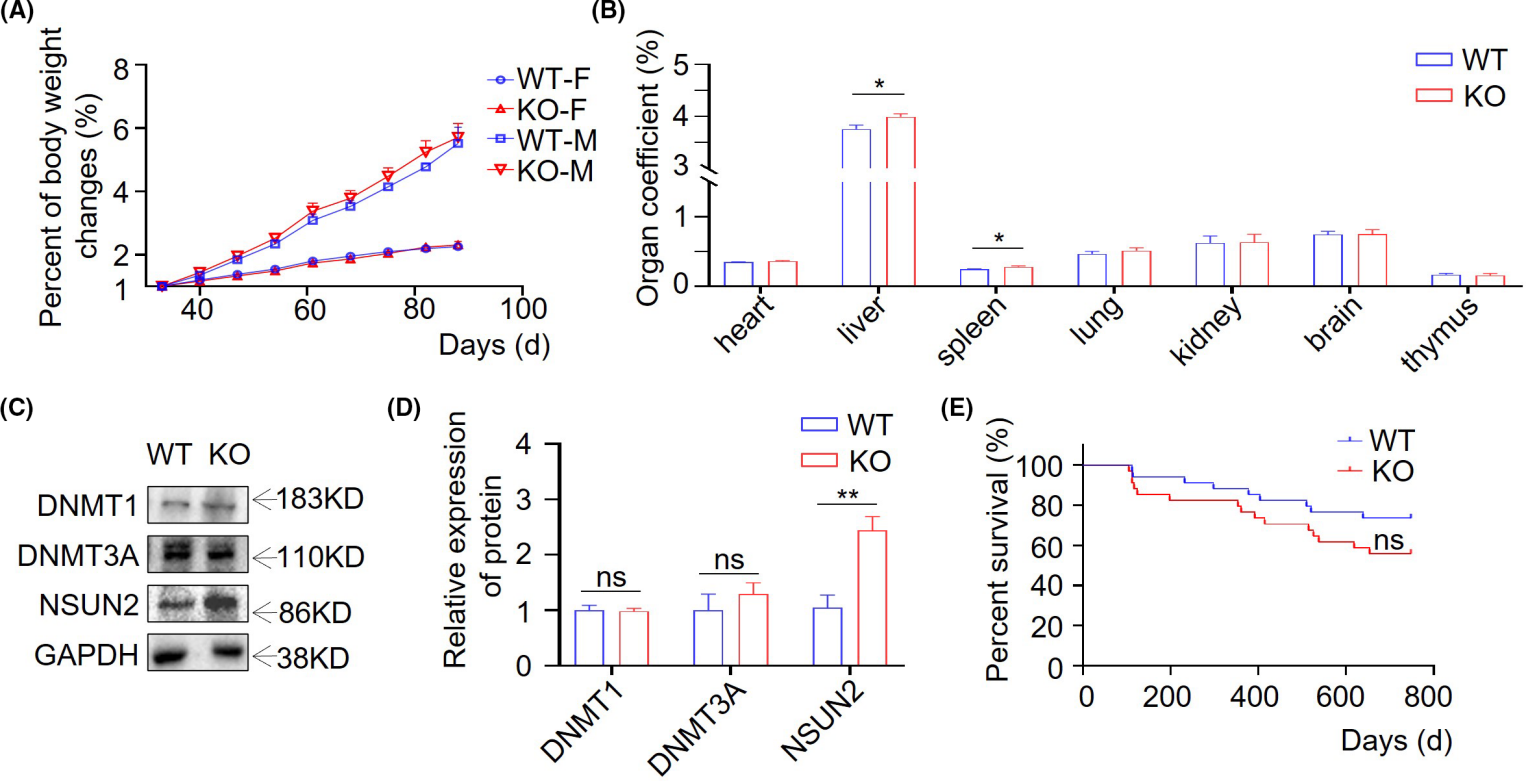
The effect of *Trdmt1* deletion on rat development and survival. A, Body weight changes of *Trdmt*1 gene knockout male and female rats (*n* = 3). F, female; M, male. B, The organ coefficient of 8‐10‐week‐old *Trdmt1* gene knockout rats (*n* ≥ 5). C‐D, Protein level of DNMT1, DNMT3A, and NSUN2 in liver analyzed by western blot of *Trdmt1* knockout rats. Quantitative analysis of DNMT1, DNMT3A, and NSUN2 protein expression level was analyzed by Image Lab software and using GAPDH for normalization. E, Survival curves of *Trdmt1* gene knockout rats (*n* = 34). WT, wild‐type rats; KO, *Trdmt1* knockout rats. Mean ± SEM; ns, not significant; *P < 0.05

It has been reported that *Trdmt1* knockout mice are viable and fertile.^10^ Here, we monitored the development and growth of *Trdmt1* knockout rats, including body weight change, organ coefficient, and lifespan. We found that the *Trdmt1* knockout homozygous ratio accorded with Mendel's law (Table S4). The body weight changes in the absence of *Trdmt1* during growth were comparable to those of WT in both female and male rats (Figure 2A). Organ coefficient analysis showed a slightly increased liver and spleen ratio in 8‐10‐week‐old knockout rats (Figure 2B). Moreover, we performed routine blood examination, with the results indicating normal blood cell count in *Trdmt1* knockout rats (Table S5). In line with these findings, flow cytometry analysis revealed that the loss of *Trdmt1* did not impair hematopoiesis, at least in 8‐10‐week‐old rats (Figure S1). To test possible compensations in *Trdmt1* knockout rats, we measured the protein expression of DNMT1, DNMT3A, and NSUN2 (Figure 2C,D). DNMT1 and DNMT3A are members of the DNMT family. NSUN2 shares a similar function with TRDMT1 and methylates C34, C48, C49, and C50 of tRNA.^37^ Surprisingly, the expression of NSUN2, but not other DNMTs, was significantly elevated in the *Trdmt1* knockout rats (Figure 2C,D). *Trdmt1* ablation had negligible impact on the growth and development of adult rats at steady state. Interestingly, we observed a minor but insignificant decrease of survival rate in *Trdmt1* knockout rats (Figure 2E), which suggests that *Trdmt1* deletion makes rats slightly more susceptible to aging.

### 3.3 *Trdmt1* deficiency resulted in increased mortality upon LPS treatment

**FIGURE 3:**
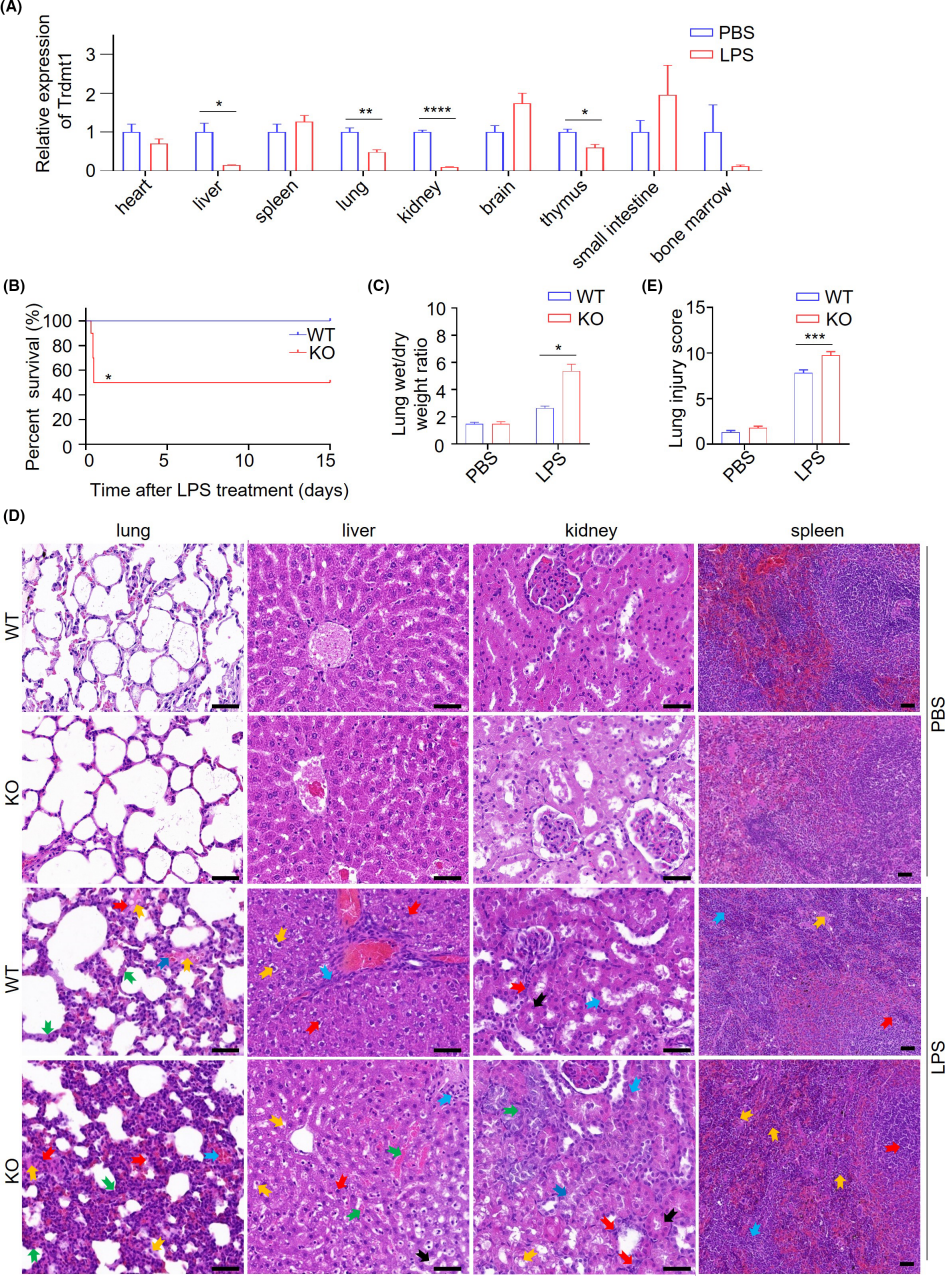
*Trdmt1* deletion leads to impaired response after LPS treatment in rats. A, The relative expression of *Trdmt1* in LPS‐treated rats in heart, liver, spleen, lung, kidney, brain, thymus, small intestine, and bone marrow tissues (*n* = 3). B, Survival curve of *Trdmt1* knockout rats after 5 mg/kg LPS treatment (*n* = 10). C, Lung wet/dry weight ratio of *Trdmt1* knockout rats after 5 mg/kg LPS treatment (*n* ≥ 3). D, Representative images of HE staining of lung, liver, kidney, and spleen from *Trdmt1* knockout rats with 1 mg/kg LPS or PBS treatment. In liver, labels indicate vacuolization of liver lobules (yellow arrow), infiltration of inflammatory cells (blue arrow), nuclei color change (red arrow), bleeding (green arrow), and hepatocyte degeneration (black arrow) in LPS‐treated groups; in lung tissue, labels indicate alveolar congestion (yellow arrow), alveolar hemorrhage (blue arrow), neutrophil infiltration (red arrow), and thickness of alveolar wall/hyaline membrane formation (green arrow) in LPS‐treated groups; in spleen tissue, labels indicate extramedullary hematopoietic foci (yellow arrow), splenic corpuscle in red pulp (blue arrow), and marginal zone thinning (red arrow) in LPS‐treated groups; in kidney, labels indicate vacuolization (yellow arrow), dilatation of renal tubules (blue arrow), neutrophil infiltration (red arrow), degeneration of renal tubules (green arrow), and tubular thickening (black arrow) in LPS‐treated groups. Scale bars, 50 μm. (E) Lung injury score of *Trdmt1* knockout rats after 1 mg/kg LPS treatment (*n* = 20). WT, wild‐type rats; KO, *Trdmt1* knockout rats. Mean ± SEM; *P < 0.05, **P < 0.01, ***P < 0.001

Growing evidence illustrates that *Trdmt1* plays a potential role in stress stimulation.^13^, ^19^, ^21^, ^22^, ^38^ Here, we applied the LPS‐induced sepsis model to analyze *Trdmt1* gene function. We first measured the expression of *Trdmt1* in different tissues after administering SD rats with LPS. We observed significantly decreased *Trdmt1* expression in liver, lung, kidney, and thymus (Figure 3A), suggesting a key role of *Trdmt1* in LPS resistance. Then, we monitored the survival rate of *Trdmt1* knockout rats in 5 mg/kg LPS‐induced sepsis model. Our data showed that the *Trdmt1* knockout group experienced a dramatic increase in mortality (Figure 3B). At the same time, this phenotype recurred in cecal ligation and puncture (CLP)‐induced inflammation rat model (Figure S2a).^39^ Thus, we came to the conclusion that *Trdmt1* is essential to the stress response.

### 3.4 *Trdmt1* deletion aggravated multiple organ damage in inflammation

From the routine blood test, we did not observe a significant difference in blood cell function between wild‐type and knockout rats after LPS treatment (Table S5). It has been reported that LPS‐induced damage mainly occurs in liver, lung, spleen, and kidney.^40^, ^41^, ^42^, ^43^, ^44^ On autopsy, we observed obviously increased scattered bleeding points and flaky bleeding foci on the fresh lung surface in *Trdmt1* knockout rats (Figure S2b). We also observed increased lung wet/dry weight (W/D) ratio in *Trdmt1* knockout rats (5.37 ± 1.41) compared with negative control (2.64 ± 0.26) (Figure 3C). On further pathological section examination, we observed an increase in the frequency of liver lobule vacuolation and hepatocyte degeneration, and lung injury score increased considerably (Figure 3E). Moreover, extramedullary hematopoietic foci increased in red pulp, and dilation and degeneration of renal tubules was more remarkable (Figure 3D). Collectively, these data reflect more severe damage of these tissues in *Trdmt1* knockout rats than their littermates. Then we explored the protein expression of DNMT1, DNMT3A, and NSUN2 in knockout rats and control rats with LPS treatment (Figure S2c‐f). Our data showed decreased expression of DNMT1, DNMT3A, and NSUN2 in LPS‐treated gene knockout rats and control rats.

### 3.5 Knockout of *Trdmt1* dramatically elevated the TNF‐α level in sepsis model

**FIGURE 4:**
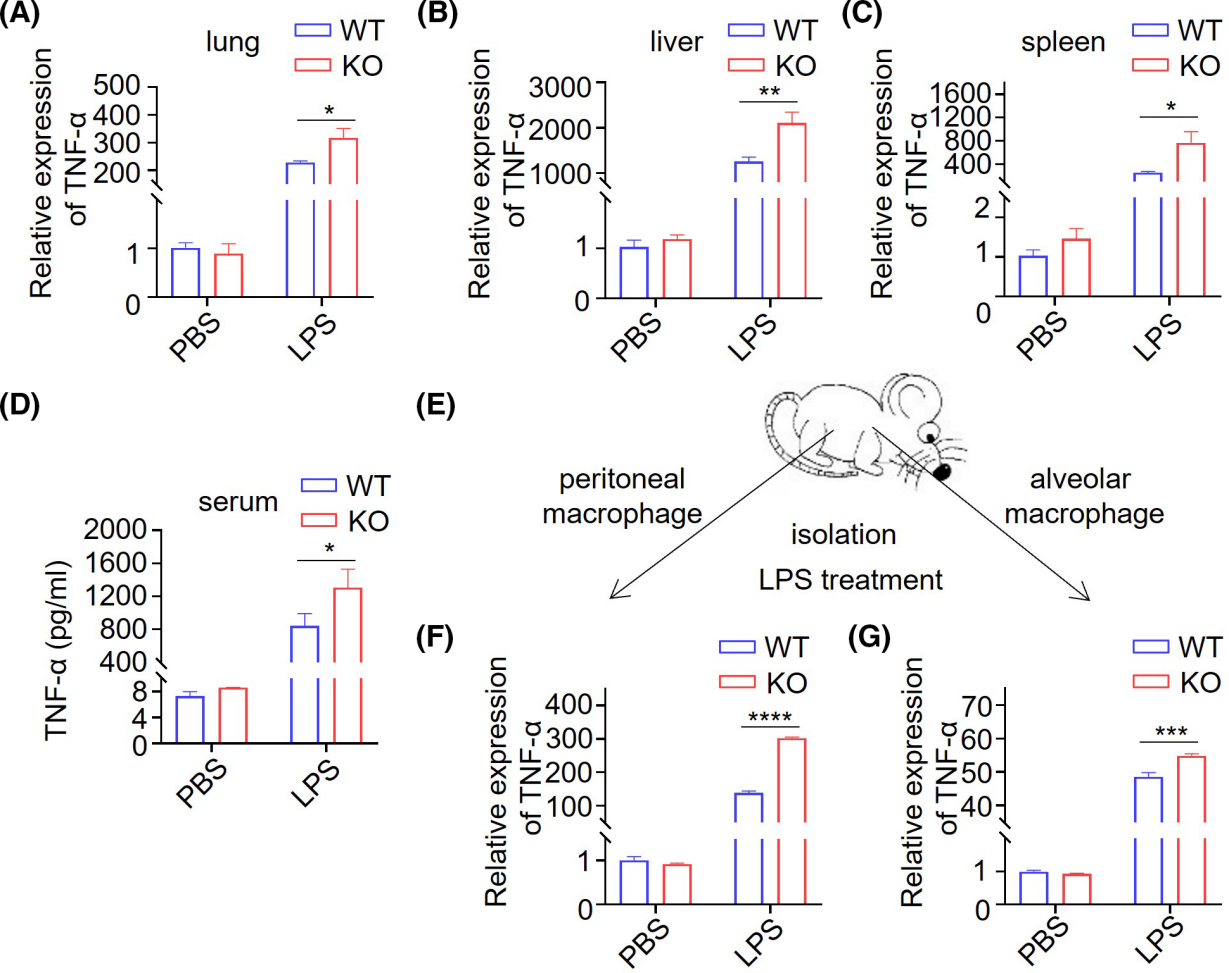
*Trdmt1* deletion leads to impaired inflammatory factor response after LPS treatment. A‐C, The relative expression of TNF‐α in lung (A), liver (B), and spleen (C) of 1 mg/kg LPS‐treated *Trdmt1* knockout rats. D, The TNF‐α protein level in serum of 1 mg/kg LPS‐treated *Trdmt1* knockout rats. E‐H, Alveolar macrophage and peritoneal macrophage derived from *Trdmt1* knockout rats were cultured and treated with 100 ng/mL LPS. The relative expression of TNF‐α of peritoneal macrophage (F), and alveolar macrophage (G) was determined. WT, wild‐type rats; KO, *Trdmt1* knockout rats. Mean ± SEM; *P < 0.05, ***P < 0.001, ****P < 0.0001

Giving that changes in inflammatory factors, including TNF‐α, IL‐1β, IL‐6, IL‐10, and iNOS, are a major characteristic of the sepsis model,^45^, ^46^ we measured the inflammatory cytokines after LPS treatment. We observed changes in inflammatory cytokines at different timepoints after LPS treatment (S3). Remarkably, the TNF‐α level was strikingly elevated in both mRNA and protein levels in the sepsis model when *Trdmt1* was deleted (Figure 4A‐D). In addition, the expression of IL‐1β was elevated at 1.5 hours after LPS treatment (Figure S3a‐c), while IL‐6 level increased at the 2‐hour timepoint (Figure S3a‐c). Decreased IL‐10 and iNOS levels were observed at the 2‐hour timepoint after LPS treatment in *Trdmt1* knockout rats (Figure S3a‐c). We further analyzed the TNF‐α level of alveolar macrophage (AM) and peritoneal macrophage (PM) treated with 100 ng/mL LPS derived from *Trdmt1* knockout and control rats. As expected, a marked escalation of TNF‐α mRNA level was also detected in AM and PM derived from *Trdmt1* knockout rats (Figure 4E‐F). Our results underscore a critical role of *Trdmt1* in regulating the levels of inflammatory cytokines, especially TNF‐α in the sepsis model. The increased release of pro‐inflammatory factors and decreased release of anti‐inflammatory factors might contribute to the enhanced aggravated tissue damage in LPS‐treated *Trdmt1* knockout rats.

### 3.6 *Trdmt1* knockout enhanced the activation of TLR4 pathway after LPS treatment

**FIGURE 5:**
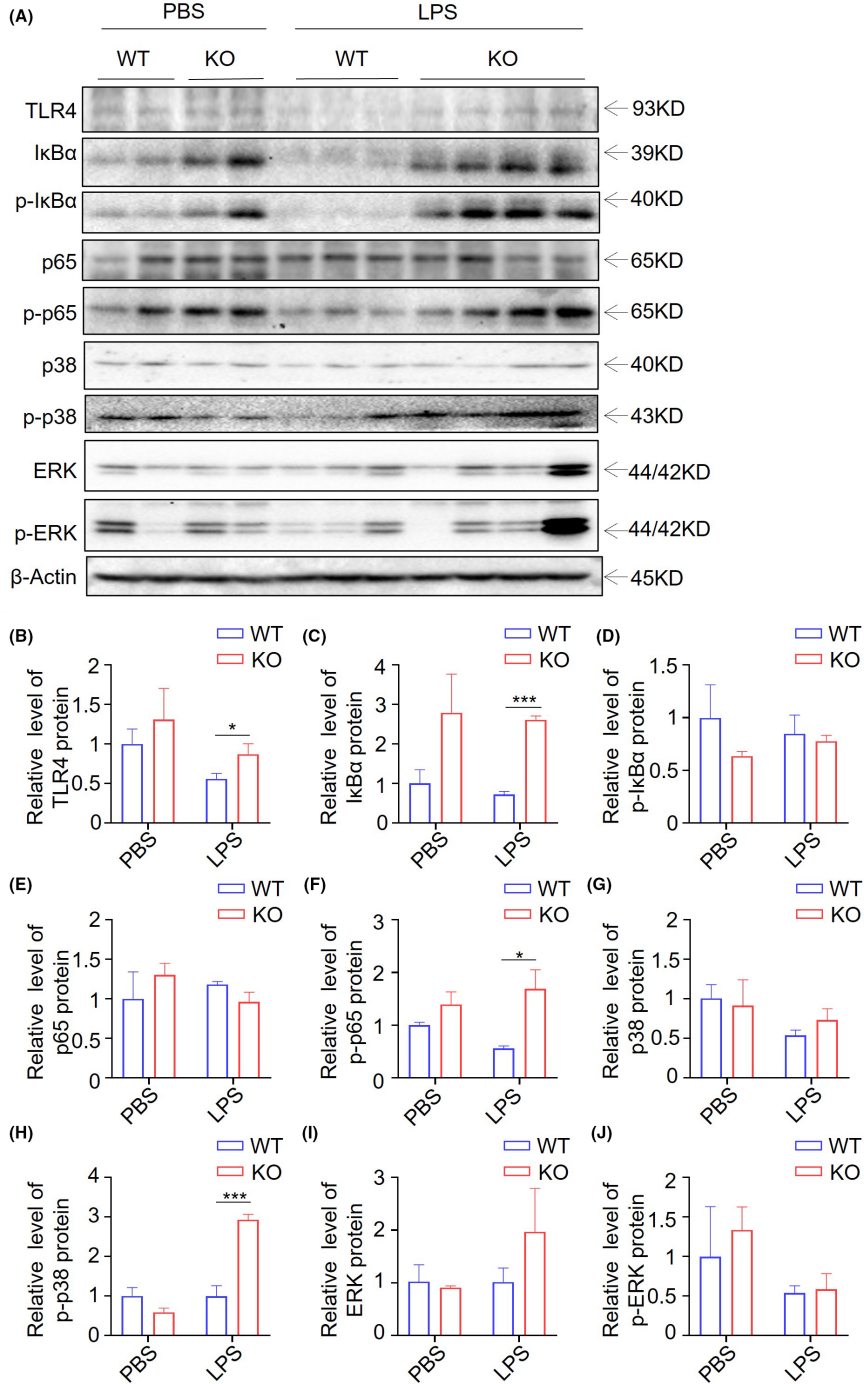
TLR4 pathway activation contributed to LPS‐induced mortality of *Trdmt1* knockout rats. A, Protein level of TLR4, IκBα, p‐IκBα, p65, p‐p65, p38, p‐p38, ERK, and p‐ERK in liver analyzed by western blot in LPS‐ or PBS‐treated *Trdmt1* knockout rats. B‐J, Quantitative analysis of TLR4 (B), IκBα (C), p‐IκBα (D), p65 (E), p‐p65 (F), p38 (G), p‐p38 (H), ERK (I), and p‐ERK (J) protein expression level analyzed by Image Lab software and using β‐Actin for normalization. WT, wild‐type rats; KO, *Trdmt1* knockout rats. Mean ± SEM; *P < 0.05, **P < 0.01, ****P < 0.0001

Considering that TLR4, but not other TLR pathways, is commonly activated in sepsis,^45^, ^47^ we performed an in‐depth analysis of the expression of TLR4 signal pathway protein. The protein level of TLR4, p‐p65, and p‐p38, but not p‐ERK, was significantly increased in liver of the LPS‐treated group upon *Trdmt1* ablation compared with WT (Figure 5A‐J). In addition, enhanced TLR4 pathway activation was also observed in spleen after *Trdmt1* knockout (Figure S4). Taken together, our findings indicate a protective role of *Trdmt1* in LPS‐induced inflammation through regulation of the TLR4 signaling pathway.

## 4 DISCUSSION

For a long time, the function of TRDMT1 has been unclear. It was originally identified from *Schizosaccharomyces pombe* as DNMT2 because it shares a catalytic function domain with de novo DNA methyltransferase DNMT1.^7^ Increasing evidence has indicated that TRDMT1 functions as a transfer RNA methyltransferase instead of a DNA methyltransferase.^10^ Though TRDMT1 depletion in zebrafish resulted in developmental perturbations, in most vertebrates TRDMT1 disruption causes no phenotypic defects.^14^, ^17^ For example, *Trdmt1*‐mutant mice were shown to be viable and fertile without visible abnormal development.^10^

In this work, we constructed *Trdmt1* knockout rats and analyzed the phenotype of *Trdmt1* knockout rats, which displayed virtually normal growth, development, reproduction, and hematopoiesis. We concluded that *Trdmt1* plays a negligible role in homeostasis under normal condition. We also assessed the possible compensations in *Trdmt1* knockout rats and observed elevated NSUN2 expression. TRDMT1 and NSUN2 are the only 2 discovered (cytosine‐5) tRNA methyltransferases to date, and they appear to play a role in tRNA stability and protein synthesis. Although lack of either TRDMT1 or NSUN2 alone has no detectable effects on mouse viability, double knockout showed a systemic lethal interaction.^48^ This indicated that the function of TRDMT1 could be compensated in part by NSUN2 in *Trdmt1* knockout rats.

Emerging evidence has suggested that *Trdmt1* is strongly correlated with stress stimulus.^13^, ^19^, ^49^ Lin et al. reported that *Trdmt1* overexpression in *Drosophila melanogaster* could extend its lifespan.^27^ In another report Li et al. found that gene expression of *Dnmt1* and *Dnmt2* (*Trdmt1*) was decreased in aging rats.^25^ These clues link the *Trdmt1* to aging, but little is known about the underlying mechanism. Here, we found the survival rate of *Trdmt1*‐deficient rats slightly but insignificantly decreased compared with their negative control, suggesting that *Trdmt1*‐deficient rats are vulnerable to environment or stress.

*Trdmt1* played a protective role against heat‐shock‐stress‐induced tRNA cleavage by transfer RNA methylation.^13^ Further study demonstrated that *Trdmt1* deletion led to increased tRNA fragmentation (tRFs) that competed with endogenous small interfering RNAs (siRNAs) to interact with Dicer‐2.^49^ In another report, *Trdmt1* participated in RNA processing during cellular stress by shuttling from the nucleus into the cytoplasm to interact with stress granules and RNA processing bodies.^19^ Inflammation is a systemic and broad stress response. However, whether *Trdmt1* functions in inflammation progression remains unknown. In the present study, we concentrated on the relationship between *Trdmt1* and inflammation. LPS has toxic effects and is widely used as a potent stimulus of inflammation,^4^ In addition, dysregulation of inflammation to LPS treatment can result in sepsis,^5^, ^6^ which places a substantial burden on the public owing to its high morbidity and mortality. It should be pointed out that LPS‐induced inflammation is easy to control and reproducible. Surprisingly, *Trdmt1* knockout significantly decreased the survival rate of rats and caused more aggravated multiple organ damage in both LPS‐treated model and CLP model. Why? We analyzed the expression of NSUN2 and DNMTs family members. The expression of DNMT1 and DNMT3A is comparable in both *Trdmt1* knockout and control rats. The expression of DNMT1 was repressed in both WT and *Trdmt1* knockout rats after LPS treatment. This is consistent with a previous report.^50^ The expression of NSUN2 in LPS‐treated *Trdmt1* knockout and WT rats is comparable, but the elevated NSUN2 expression in LPS‐treated *Trdmt1* knockout rats was repressed.

Inflammation imbalance represents the basis of the sepsis pathogenesis and is sustained throughout the process of sepsis.^51^ Infectious agents induce acute responses, including macrophages activation and pro‐inflammatory cytokine release, further forming a cytokine storm.^52^ This process is mediated by diverse pattern‐recognition receptors (PRRs), including the most‐studied Toll‐like receptors (TLRs), through damage‐associated molecular patterns (DAMPs) or pathogen‐associated molecular patterns (PAMPs).^53^ TLRs interact with their ligands and activate several signaling pathways, including mitogen‐activated protein kinase (MAPK) and nuclear factor‐κB (NF‐κB), to produce inflammatory cytokines such as interleukin (IL)‐1, IL‐6, and tumor necrosis factor‐α (TNF‐α).^45^, ^54^, ^55^, ^56^, ^57^ We observed increased release of pro‐inflammatory factors and decreased release of anti‐inflammatory factors release at different timepoints. More importantly, TNF‐α level was remarkably elevated in the *Trdmt1* knockout rat liver, lung, and spleen tissues as well as in AM and PM. Moreover, in K562 cells, we found that overexpression of *Trdmt1* resulted in lower level of TNF‐α after LPS treatment (data not shown). This phenotype was in accordance with our results presented in this study. Further, we explored the underlying mechanism and found increased phosphorylation levels of NF‐κB and p38 in *Trdmt1* knockout rats. This indicates enhanced TLR4 signal pathway activation. Even though the direct relationship between TRDMT1 and TLR4 signal remains unclear, our data highlight *Trdmt1* as playing a protective role in the inflammation process through the TLR4‐NF‐κB/MAPK‐TNF‐α signaling pathway.

In summary, we proved for the first time the function of *Trdmt1* in inflammation. Specifically, *Trdmt1* was required to survive inflammation stress through the NF‐κB/MAPK‐TNF‐α signaling pathway. Indeed, our work provides useful information to extend our understanding of *Trdmt1* function under stress conditions, especially inflammation. This work may be helpful for patients with sepsis in clinic treatment.

## CONFLICT OF INTEREST

The authors declare no conflict of interest.

## AUTHOR CONTRIBUTIONS

Y.M. conceived the project and supervised the research. X.Q. and Z.L. designed all the experiments, performed the experiments, and analyzed the data with the assistance of X.Z., L.Y., W.K., W.C., W.D., L.L., D.L., L.G., and L.Z. Y.M., X.Q., and Z.L. wrote the manuscript, data analysis, and paper discussion.

## Supporting information


Data S1
