# Supplementary material for: TRDMT1 exhibited protective effects against LPS‐induced inflammation in rats through TLR4‐NF‐κB/MAPK‐TNF‐α pathway
Source: Animal Model Exp Med. 2022 Apr 27;5(2):172–82. doi: 10.1002/ame2.12221 (PMC9043724; doi:10.1002/ame2.12221)
Supplement: Supplementary file 1 — Data S1 [file AME2-5-172-s001.docx]

**Title:** TRDMT1 exhibited protective effects against LPS-induced inflammation in rats through TLR4-NF-κB/MAPK-TNF-α pathway

**Running title:** TRDMT1 exhibited protective effects in LPS-induced inflammation

Zhengguang Li^1,2,4^, Xiaolong Qi^2,3,4^, Xu Zhang^1,2^, Lei Yu^1,2^, Lijuan Gao^1,2^, Weining Kong^2,3^, Wei Chen^1,2^, Wei Dong^2,3^, Lijun Luo^1,2^, Dan Lu^1,2^, Lianfeng Zhang^1,3^, Yuanwu Ma^1,2,3*^

^1^Key Laboratory of Human Disease Comparative Medicine, National Health Commission of China (NHC), Institute of Laboratory Animal Science, Chinese Academy of Medical Sciences, Peking Union Medicine College, Beijing 100021, China.

^2^National Human Diseases Animal Model Resource Center and Beijing Engineering Research Center for Experimental Animal Models of Human Critical Diseases, Institute of Laboratory Animal Science, Peking Union Medicine College, Chinese Academy of Medical Sciences, Beijing 100021, China.

^3^Neuroscience center, Chinese Academy of Medical Sciences, Beijing 100730, China.

^4^These authors contributed equally to this work.

***** Correspondence: [mayuanwu@cnilas.org](mailto:mayuanwu@cnilas.org); Tel: +8610-6777 6394; Address: #5 Panjiayuan Nanli, Chaoyang District, Beijing, China.

**Supplementary Data** including Supplementary Methods, Supplementary Figures, and Supplementary Tables.

**SUPPLEMENTARY METHODS**

**Blood routine examination**

Two hundred microliter rat orbital blood was collected into 1.5 ml tube containing 50 μL PE (PBS contained 2 mM EDTA) ^1^. The blood samples were measured and analyzed using XN-1000 V hematology analyzer (Sysmex).

**Flow cytometry analysis**

Single-cell suspensions of bone marrow (BM), peripheral blood (PB), spleen and thymus were prepared as previously described.^2^ The CD4/CD3/CD8a/CD45RA/CD161/Gra/Mac/Thy1.1 antibodies were used for flow cytometric analysis and the detailed information was provided in Table S6. Samples were detected using a BD LSR Frotessa (BD Biosciences, San Jose, CA, USA) and data was analyzed using FlowJo10.0.7 software (TreeStar, San Carlos, CA, USA).

**CLP-induced sepsis model**

Eight to ten-week old SD rats were anesthetized via isoflurane, and the abdomen was shaved with a longitudinal abdominal midline incision (3 to 4 cm). After dissection of the mesentery of the cecum, a ligature was placed around the cecum at the designated position and ligated to occlude the cecal lumen at the point. The cecum was placed back into abdominal cavity and wound was sutured. Next, 0.3 ml of saline was administered subcutaneously for fluid resuscitation. Rats were returned to their home cage. Likewise, the survival was monitored.

**SUPPLEMENTARY FIGURES**


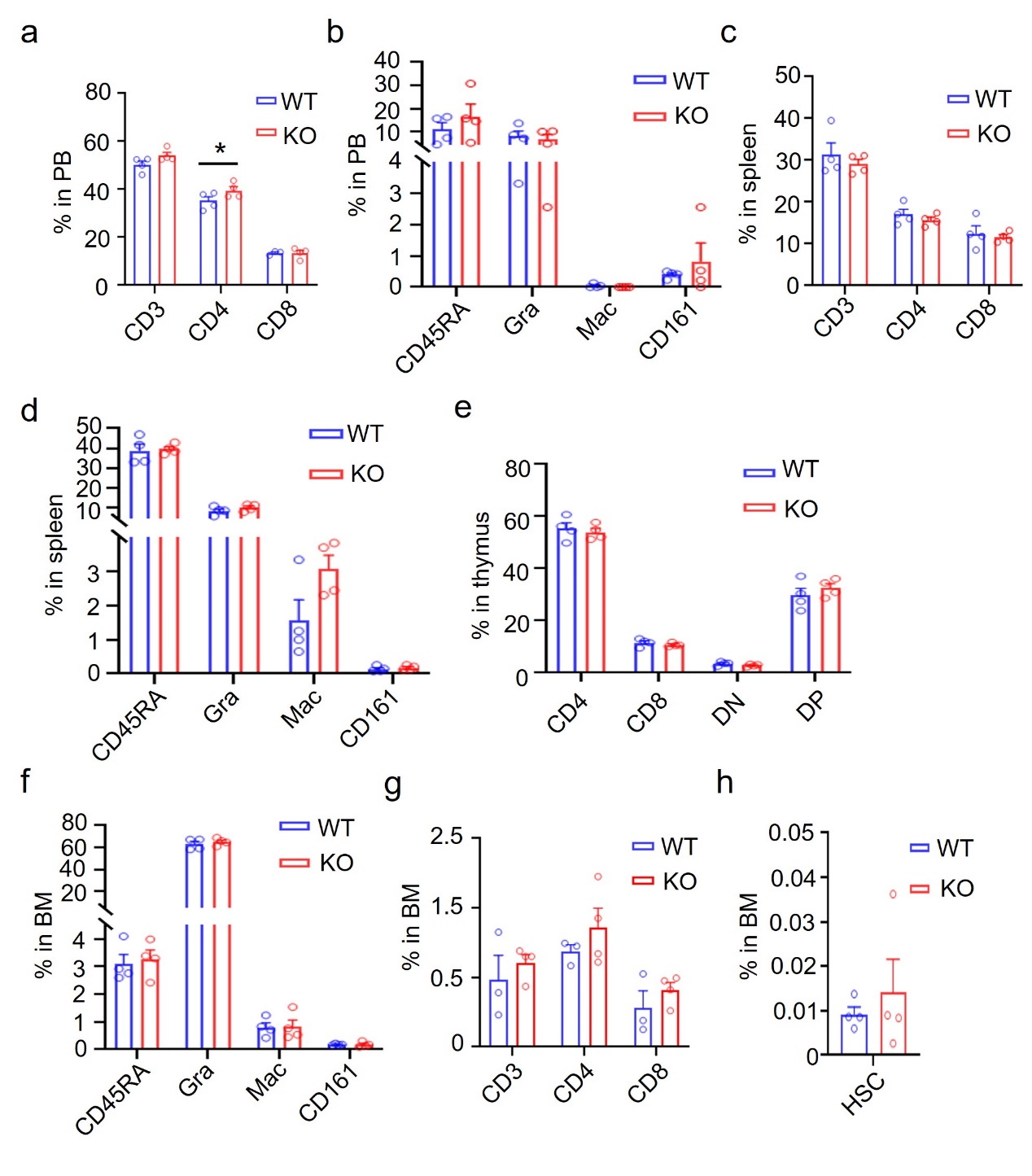


**Figure S1 The impact of *Trdmt1* deletion on hematopoiesis**

a. FACS analysis the frequencies of T cells in peripheral blood (PB). b. FACS analysis the frequencies of CD45RA^+^, CD161^+^, Mac^+^, and Gra^+^ cells in PB. c. FACS analysis the frequencies of T cells in spleen. d. FACS analysis the frequencies of CD45RA^+^, CD161^+^, Mac^+^, and Gra^+^ cells in spleen. e. FACS analysis the frequencies of T cells in thymus. f. FACS analysis the frequencies of CD45RA^+^, CD161^+^, Mac^+^, and Gra^+^ cells in bone marrow (BM). g. FACS analysis the frequencies of T cells in BM. h. FACS analysis the frequencies of hematopoietic stem cell (HSC) cells in BM. WT: wildtype rats; KO: *Trdmt1* knockout rats. Mean ± SEM; * P <0.05.


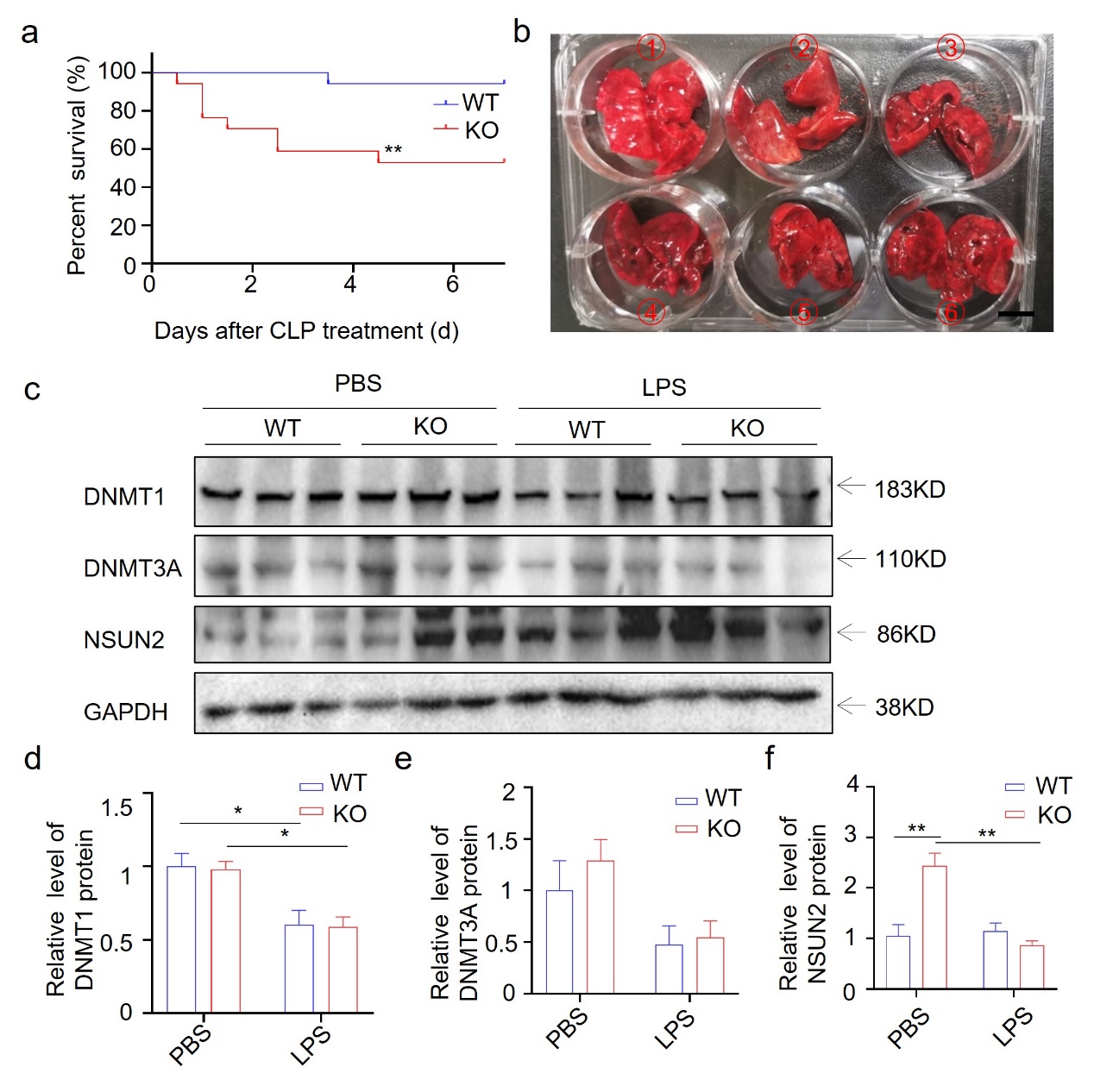


**Figure S2 *Trdmt1* deletion leads impaired response in sepsis model**

a. Survive curve of *Trdmt1* knockout and wild-type rats after cecal ligation and puncture (CLP) treatment (n=17). b. Photograph of LPS treated fresh lung. 1,2 for wild type rats; 3-6 for *Trdmt1* knockout rats. c. Protein level of DNMT1, DNMT3A and NSUN2 were analyzed by western blot in liver of LPS or PBS treated wildtype and *Trdmt1* knockout rats. Quantitative analysis of DNMT1 (d), DNMT3A (e), and NSUN2 (f) proteins expression level were analyzed by image lab software and using GAPDH for normalization. WT: wildtype rats; KO: *Trdmt1* knockout rats. Mean ± SEM; * P <0.05, ** P <0.01.


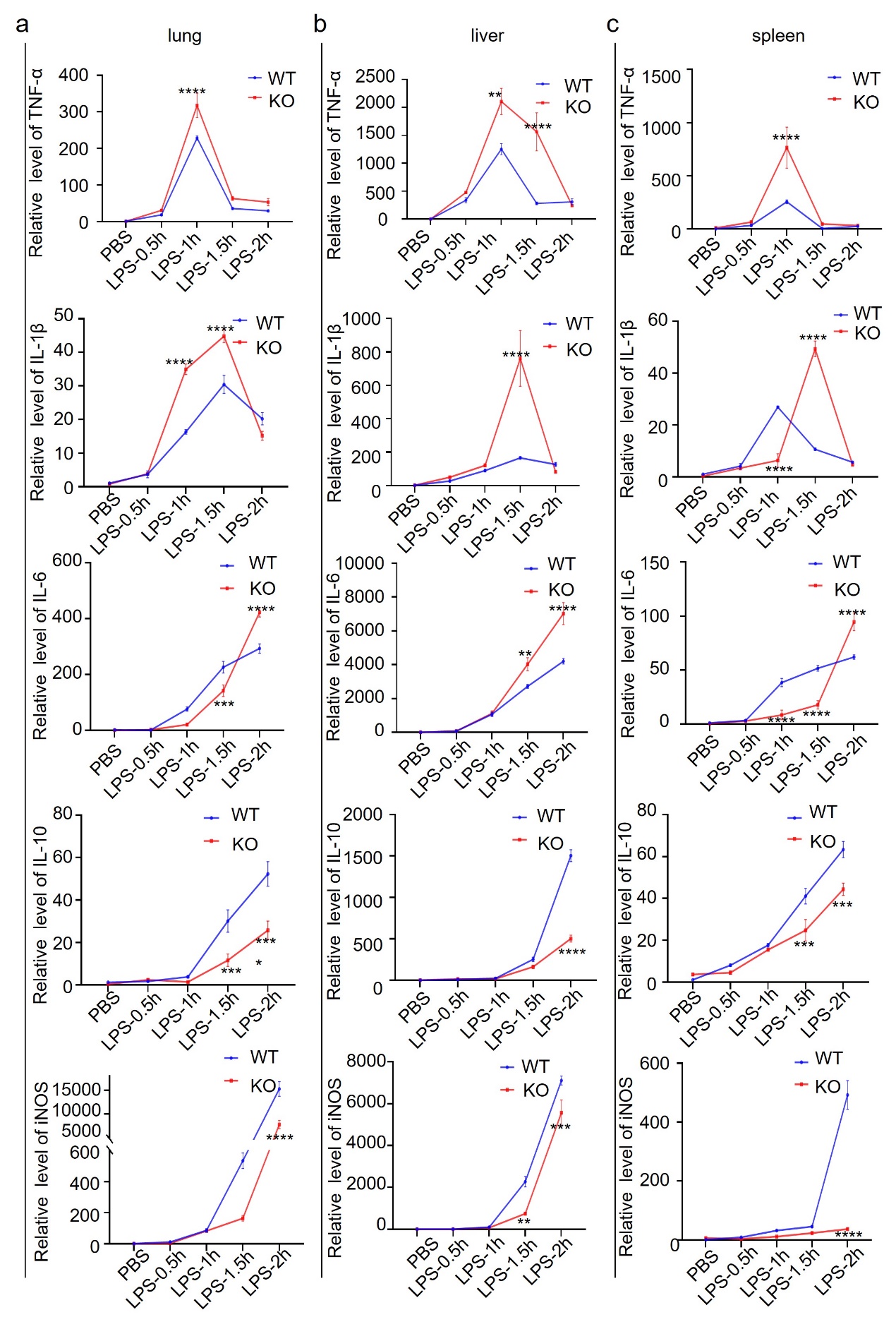


**Figure S3 Inflammatory factor changes after LPS treatment in *Trdmt1* knockout rats**

a. The relative expression of TNF-α, IL-1β, IL-6, IL-10 and iNOS was detected in lung (a), liver (b) and spleen (c) of *Trdmt1* knockout rats which were treated with PBS or LPS for 0.5h, 1h, 1.5h and 2h using real-time PCR. WT: wildtype rats; KO: *Trdmt1* knockout rats. Mean ± SEM; ** P < 0.01, *** P < 0.001, **** P < 0.0001.


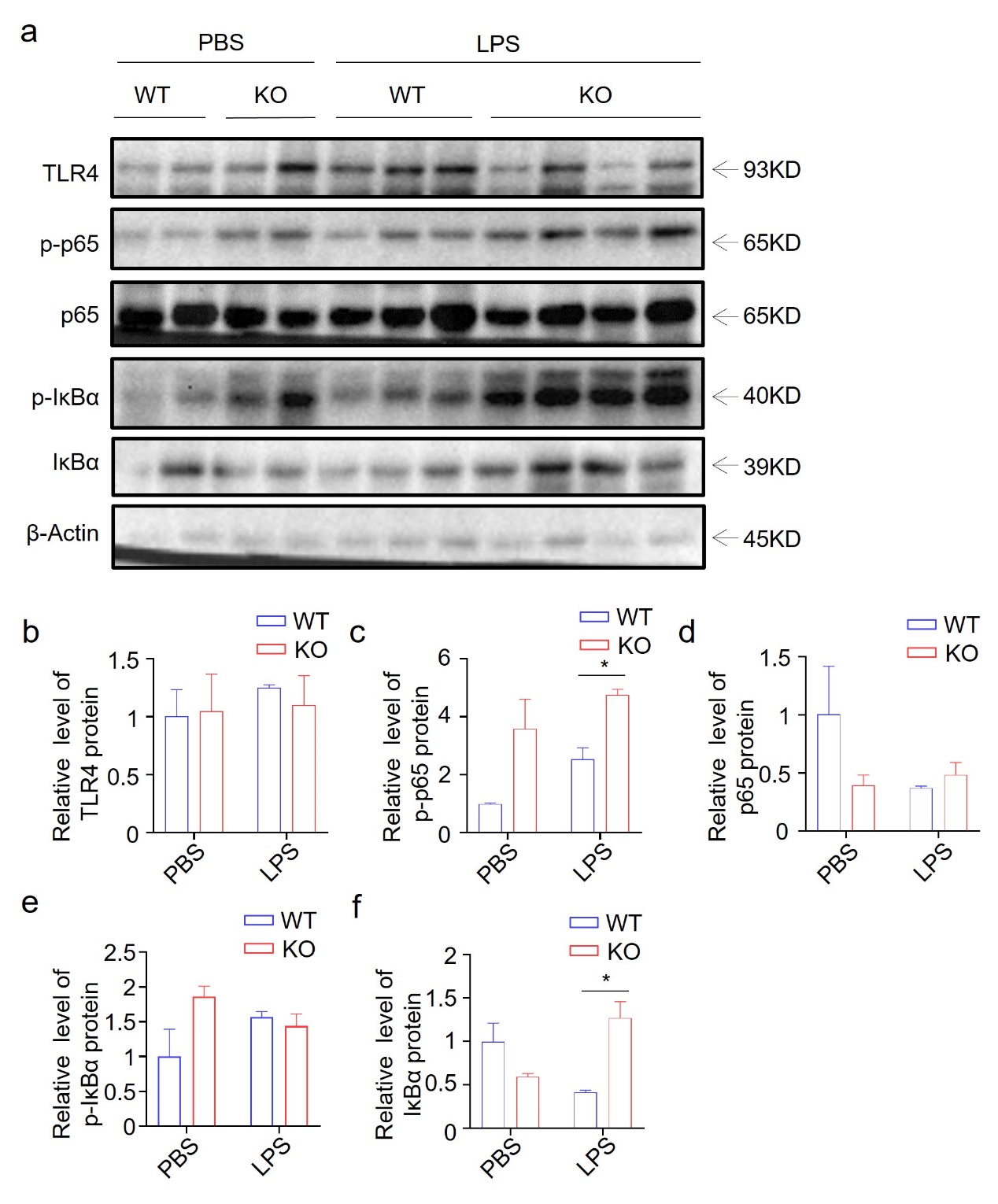


**Figure S4 TLR4 signal pathway activation also detected in spleen tisusse**

a. Protein expression of TLR4, p-p65, p65, p-IκBα, and IκBα in spleen were detected by Western blot in LPS or PBS treated *Trdmt1* knockout rats. Quantitative analysis of TLR4 (b), p-p65 (c), p65 (d), p-IκBα (e), and IκBα (f) proteins expression using β-Actin for normalization. WT: wildtype rats; KO: *Trdmt1* knockout rats. Mean ± SEM; * P < 0.05, ** P < 0.01, *** P < 0.001.

**SUPPLEMENTARY TABLES**

**Table S1 oligos used for sgRNA preparation**

| oligos | Sequence (5’-3’) |
| --- | --- |
| Rat-*Trdmt1*- -gRNA1 up | TAGGATAACTACATTTCAAGAT |
| Rat-*Trdmt1*- -gRNA1 down | AAACATCTTGAAATGTAGTTAT |
| Rat-*Trdmt1*- -gRNA2 up | TAGGCTCAGTACTGCTCCCAGT |
| Rat-*Trdmt1*- -gRNA2 down | AAACACTGGGAGCAGTACTGAG |

**Table S2 Primers used for genotyping of *Trdmt1* knockout rats**

| Primers | Sequence (5’-3’) |
| --- | --- |
| *Trdmt1*-P1 | CAAATGAACCCAAGTTGCTTC |
| *Trdmt1*-P2 | TCCTGTTTGCTAAGTAACTACAAGTGC |
| *Trdmt1*-P3 | ATGCACACACTTATTATAAATCCTACAG |
| *Trdmt1*-P4 | TCTCACAATGCAGGTCCACT |

**Table S3 Primers used for gene relative expression detection using real-time PCR**

| Primers | Sequence (5’-3’) |
| --- | --- |
| r-*Trdmt1*-RT-F1: | CCAAACTCAAGGCTACGGTATT |
| r-*Trdmt1*-RT-R1: | CTGTCCCTTCTATGTAGCTTCCAT |
| r-*actin*-F | ACCCGCCACCAGTTCGC |
| r-*actin*-R | CACGATGGAGGGGAAGACG |
| r-*IL1β*-F | CTGTGACTCGTGGGATGATG |
| r-*IL1β*-R | GGGATTTTGTCGTTGCTTGT |
| r-*IL6*-F | CCGGAGAGGAGACTTCACAG |
| r-*IL6*-R | ACAGTGCATCATCGCTGTTC |
| r-*TNFα*-F | AGATGTGGAACTGGCAGAGG |
| r-*TNFα*-R | CCCATTTGGGAACTTCTCCT |
| r-*iNOS*-F | AGGGAGTGTTGTTCCAGGTG |
| r-*iNOS*-R | TCCTCAACCTGCTCCTCACT |
| r-*IL10*-F | CCTGCTCTTACTGGCTGGAG |
| r-*IL10*-R | TGTCCAGCTGGTCCTTCTTT |
| r-*GAPDH*-F | CTCATGACCACAGTCCATGC |
| r-*GAPDH*-R | TTCAGCTCTGGGATGACCTT |

**Table S4 Summary of birth rate of *Trdmt1* knockout homozygous and heterozygous**

| homozygous | heterozygous | WT | Total |
| --- | --- | --- | --- |
| 168(49.53%) | 313(26.58%) | 151(23.89%) | 632 |

**Table S5 Blood routine result of *Trdmt1* knockout rats in LPS stress and not-treated control**

| Blood routine | WT-NC | KO-NC | P value | WT-LPS | KO-LPS | P value |
| --- | --- | --- | --- | --- | --- | --- |
| Leukocyte | | | | | | |
| Total WBC(10^9^/L) | 1.42±0.91 | 1.68±1 | 0.5844 | 1.35±0.93 | 1.69±1.05 | 0.5611 |
| Neutrophils(10^9^/L) | 0.57±0.41 | 0.57±0.34 | 0.994 | 0.4±0.16 | 0.85±0.49 | 0.1367 |
| Lymphocytes(10^9^/L) | 0.74±0.82 | 1.92±1 | 0.0596 | 0.48±0.81 | 0.36±0.23 | 0.8601 |
| Monocytes(10^9^/L) | 0.05±0.05 | 0.05±0.03 | 0.7553 | 0.07±0.03 | 0.14±0.11 | 0.3399 |
| NE% | 21.5±6.33 | 22.99±7.09 | 0.6917 | 39.3±11.97 | 63.2±16.64 | 0.0585 |
| LY% | 62.24±11.5 | 68.78±9.34 | 0.2462 | 16.05±8.41 | 20.35±0.21 | 0.5451 |
| MO% | 3.73±2.07 | 2.58±1.86 | 0.293 | 7.1±2.19 | 7.15±1.2 | 0.979 |
| Erythriod/megakaryocytic | | | | | | |
| RBC(10^12^/L) | 6.01±0.65 | 6.2±0.48 | 0.5131 | 5.65±0.5 | 5.84±0.63 | 0.565 |
| HGB(g/L) | 10.7±0.58 | 10.78±0.67 | 0.8076 | 9.9±0.81 | 10.16±0.8 | 0.5937 |
| HCT(%) | 36.47±2.26 | 37.76±3.03 | 0.3302 | 34.5±2.71 | 36.42±3.55 | 0.3114 |
| MCV(fL) | 60.93±3.71 | 61.04±5.12 | 0.962 | 61.14±2.26 | 62.38±2.49 | 0.3908 |
| MCH(pg) | 17.88±1.1 | 17.44±1.14 | 0.4304 | 17.54±0.55 | 17.46±0.62 | 0.8112 |
| MCHC(g/dL) | 29.34±0.53 | 28.6±0.77 | *0.0334 | 28.73±0.57 | 28.02±0.73 | 0.0861 |
| RDW(%) | 12.64±0.51 | 12.44±0.43 | 0.3852 | 14.26±0.44 | 14±0.78 | 0.4808 |
| HDW(g/dL) | 2.57±0.28 | 2.53±0.36 | 0.757 | 2.68±0.15 | 2.71±0.3 | 0.7983 |
| Platelets(10^9^/L) | 933.44±111.34 | 835.25±166.35 | 0.1687 | 803.86±238.8 | 821±316.52 | 0.9166 |
| MPV(fL) | 9.6±0.5 | 9.56±0.41 | 0.8687 | 9.37±0.51 | 9.84±1.07 | 0.3314 |

|  |  |
| --- | --- |

**Table S6 Antibodies used in flow cytometry and western blot**

| Antibody | Origin | Cat. No | Assay |
| --- | --- | --- | --- |
| FITC-CD4 | eBioscience | 11-0040-82 | FC |
| FITC-Gra | eBioscience | 11-0570-82 | FC |
| PE-CD3 | eBioscience | 12-0030-82 | FC |
| Pecy7-CD8a | eBioscience | 25-0084-82 | FC |
| APC-eFluor780-SA | eBioscience | 47-4317-82 | FC |
| APC-CD45RA | eBioscience | 17-0462-82 | FC |
| Percp-cy5.5-Thy-1.1 | eBioscience | 45-0900-82 | FC |
| CD161-biotin | eBioscience | MA5-17537 | FC |
| PE-Mac | eBioscience | 12-0660-82 | FC |
| TRDMT1 | Santa Cruz | sc-365001 | WB |
| DNMT1 | invitrogen | TL2680927 | WB |
| DNMT3A | invitrogen | TK2671326B | WB |
| NSUN2 | proteintech | 20854-1-AP | WB |
| p65 | CST | 8242S | WB |
| p-p65 | CST | 3033S | WB |
| p38 | CST | 8690S | WB |
| p-p38 | CST | 4511S | WB |
| TLR4 | Abcam | ab13556 | WB |
| IκBα | CST | 4814S | WB |
| p-IκBα | CST | 9246S | WB |
| ERK | CST | 9102S | WB |
| p-ERK | CST | 4370S | WB |
| β-Actin | CST | 3700S | WB |

FC, flow cytometry; WB, western blot; CST, Cell Signaling Technology

**REFERENCE**

1. Guo, H., et al., *PBX3 is essential for leukemia stem cell maintenance in MLL-rearranged leukemia.* Int J Cancer, 2017. **141**(2): p. 324-335.

2. Li, M., et al., *SETD5 modulates homeostasis of hematopoietic stem cells by mediating RNA Polymerase II pausing in cooperation with HCF-1.* Leukemia, 2021.
